# Psychometric properties of the Chinese version of the Client Empowerment Scale in chronic patients

**DOI:** 10.1186/s40064-016-3183-4

**Published:** 2016-09-21

**Authors:** Chunlan Zhou, Xue Ji, Jian Tan, Yanni Wu

**Affiliations:** Nanfang Hospital, Southern Medical University, 1838 Guangzhou Rd, Guangzhou, 510515 China

## Abstract

**Objective:**

The objective of the study was to evaluate the psychometric properties of the Chinese version of the Client Empowerment Scale (CCES).

**Methods:**

In this cross-sectional survey, content validity was examined based on the reviews of a panel of five experts; test–retest was conducted to assess the item reliabilities of the scale. A convenience sample of 317 patients with chronic diseases were recruited from three level-3 hospitals were selected to explore the factorial structure of the CCES using exploratory factor analysis.

**Results:**

The Chinese Client Empowerment Scale was developed by modifying seven items. Results indicated that the CCES demonstrated good internal consistency and test–retest reliability. Principal component analysis supported the six-factor structure of the original instrument (Informed Confidence, Client–Provider Relationship, Social Advocacy, Awareness, Control and Client–Client Support) were identified and confirmed in the CCES. There were significant correlations among the six factors, which demonstrated the good construct validity of the Chinese version of this scale.

**Conclusion:**

The findings support the reliability and validity of the CCES, and the 44-item six-factor Chinese version of the CES is a self-completion scale.

## Background

China is undergoing a dramatic demographic change as an aging population increased and life expectancy grows today (Gonghuan 2008). According to epidemiological data, chronic diseases are the main cause of mortality in these societies. In line with this finding, the World Health Organization (2005) reports they account for more than 60 % of all deaths. These health conditions are defined by a long duration and gradual progression. Chronic diseases negatively affect patients’ wellbeing, cause their serious inconvenience and persist till the end of their lifetime. Also, patients with chronic diseases have to adjust to the new situation with its many limitations resulting from the character of the disease. This is a very difficult task to accomplish indeed (Aujoulat et al. 2007). Thus, the aim of health care for the chronically ill patients was not to cure the disease, but to control symptoms and ensure the quality of life.

Patient empowerment are able to provide the medical model for an available management of chronic health conditions (Kleier and Dittman 2014). In the field of health-care, empowerment has been admitted as a method in order to guide the provider–patient relationship. Whereas in the traditional health-care, patients are seen as the embracer of medical decisions and prescriptions, the empowerment approach views patients as being responsible for their decisions and the consequences of their decisions (Aujoulat et al. 2007). Within an empowerment framework, the responsibility of health-care providers is to recognize the suffering of patients, identify patients’ strengths, and prevent further marginalization of patients due to power inequality (Anderson and Funnell 2009). In the empowerment process, the patient has the right to choose their treatment. Health care providers provide clear, concise, accurate information and patients can access and discuss a range of information, options, and views to help them self-determine and self-manage their disease. The concept of patient empowerment emphasizes health care to be patient-centered, providing educational materials and decision-making aids, such as leaflets, computer programs, interactive videos, websites, and group presentations. These materials help patients identify their own skills and needs and empower themselves instead of just focusing on compliance with the offered treatments (Cyrino et al. 2009).

Patient empowerment is an effective approach to help patient change behavior and choose personal meanings (Anderson and Funnell 2010). The empowerment approach is especially appropriate in chronic diseases because the recommended behavior changes involved deeply embedded aspects of the patient’s daily life (Salmon and Hall 2003). To maximize the chance for change, patients must be internally motivated (e.g., “Exercise is really important to me.”) rather than externally motivated (e.g. “My doctor wants me to exercise.”) (Taylor 2010). In the past years since we presented the philosophy of patient empowerment as a viable approach to chronic disease care and education and a great deal has changed (Paterson 2001).

To develop a valid and reliable measure of the patient empowerment, Mikky conducted a large-scale social study (Mikky 2006). The questionnaire was posted on several online support groups designed for clients with various chronic health conditions. The study samples consisted of 318 participants worldwide, who live with chronic health condition(s), and data were stored on a secured server at www.formsite.com. Date analysis showed that the CES scale is highly reliable with Alpha (a) of 0.97. Statistical techniques to assess the construct validity of the CES scale were executed using two methods of factor analysis: principal component analysis, and principal axis factoring. After analyzing factor and reliability, they retained 44 items with the highest factor loadings for the final scale, that is, the Client Empowerment Scale. A reliable and valid CES instrument can be used to measure clients’ empowerment, predict clients’ self-management practices, and evaluate the effectiveness of empowering programs.

Although much research about empowerment are presented (Table 1) (Mikky 2006; Anderson 2003; Bulsara 2006; Faulkner 2001; Kettunen 2006; Lars and Tommy 2005; Rogers 1997), only CES can be used in clinical research to measure clients’ empowerment, predict clients’ self-management practices, and evaluate the effectiveness of empowering programs. Therefore, the objective of this study was to examine the psychometric properties of the Chinese version of the CES for patients with chronic diseases, perform across-cultural validity assessment apply this measure to patients with chronic diseases.

**Table 1:** Summarized research results of Empowerment Scale

Author(s)	Measure	Methods	Subjects	Results
Mikky	44-Item Client Empowerment Scale	Principal component analysis	318 clients with various chronic health conditions	The 44 items were rescored on a five-point scale
Rogers et al.	28-Item Empowerment Scale	Principal components factor analysis	271 members of six self-help Programs	Five-factors: self-efficacy, power, community activism; righteous anger; and optimism and control over the future
Faulkner	100-Item Patient Empowerment/Disempowerment Scale	Frequency score	102 elderly patients	Offered as a means of identifying hospital environments which facilitate independence
Anderson et al.	28-Item Diabetes-Patient Empowerment Scale	Principal component analysis diabetes	375 and 229 diabetes patients	Three-factor solution accounts for 56 % of the total variance
Bulsara et al.	28-Item Patient Empowerment Scale	Rasch model analysis	100 cancer patients	Fitted the Rasch model with the exception of two items
Hansson and Bjorkman	28-Item Empowerment Scale	Confirmatory factor analysis	176 subjects with mental illness	Good construct validity; two-factors: self-esteem and activism and community and power
Kettunen et al.	43-Item Empowering-Speech Scale	Confirmatory factor analysis	127 counseling situations	Second-order two-factor solution explained 59 % of variation

## The study

### Aim

The objective of the study was to evaluate the psychometric properties of the Chinese version of the Client Empowerment Scale (CCES).

### Design

Cross-sectional design was used. Firstly, we developed a Chinese version of CES based on Mikky’s. Secondly, we tested the psychometric properties of Chinese version of the scale.

### Participants

A convenience sampling strategy was used to recruit participants from three level-3 hospital in Guangzhou, China, during the period from July 2015 to December 2015. Participants represented multiple geographic locales in China. The eligibility participants were those who were (1) at least 1 year diagnosed with chronic disease, (2) above 18 years old, (3) without any cognitive disorders, (4) able to independently read and make decisions, and (5) willing to participate in the study. The exclusive participants were those who were (1) with acute complications, (2) restricted with visual impairment due to complications or complications, (3) with severe co-morbid mental illness, (4) or not have the ability to read and write Chinese.

According to Kendall sample estimation method, the sample size of the sample was 5–10 times of the number of variables. In this study, the questionnaire items were 44, sample size was 6 times of the questionnaire items, increase 20 % invalid response, and ultimately determine the sample size for 317 cases. So a sample of 317 participants was recruited from 317 eligible patients (participation rate of 100 %) for the study. In addition, 20 chronic patients were selected from the 317 participants for evaluation of test–retest reliability. The response rate was 100 % for the evaluation of test–retest reliability.

### Instrument

The CCES consisted of two parts. Part 1 was the general information of patients, including gender, age, education level, economic status, disease type and time since diagnosis. Part 2 was the Client Empowerment Scale. The CES was originally developed by Mikky who designed a reliable and valid measure was designed for client empowerment. The CES contains 44 items, 6 dimensions which were Informed Confidence, Client–Provider Relationship, Social Advocacy, Awareness, Control, Client–Client Support. Five-point Likert-based scale from the original author was employed, 1 means strongly disagree, 2 means disagree, 3 means uncertain, 4 means agree, 5 means strongly agree.

### Procedures

Consented by author, our study procedures were completed in several steps. These steps consisted of scale translation, culture adaptation, item extreme value analysis and item-total correlation, content validity, and reliability analysis (internal consistency and test–retest reliability) to develop the CCES. The detailed steps included the following:

*Translation* According to the principle of Brislin’s double translation and back translation (Cha et al. 2007), two professional graduate students who master English completed a forward translation of the CES from English to Chinese. Three chronic disease experts and two nursing educators make their evaluation and modification on these two copies of translated Chinese CES, and ultimately determine the Chinese version of the CES which was linguistically and conceptually match with the original English version of the CES. Second, two graduate students, blinded to the original English version, translated the Chinese CES back to English. The contents of the translation was identified by bilingual experts to establish semantic equivalence.

*Culture adaptation* We then conducted the culture adaptation process by means of a patient-expert panel approach. In order to test whether the scale was suitable for the Chinese chronic population, 30 patients with chronic diseases from Department of Cardiology, 3 chronic disease experts, 2 nursing educators, were convened to discuss the meaning of each item, giving their opinions on the cultural equivalency of the CCES and the appropriateness of the language translation. The results showed that the item of scale was easy to understand with clear semantic structure, and the patient could complete the scale between 15 and 25 min.

*Item analysis* Item-total correlation was applied to identify problematic items of the CCES. We used the item-total correlation coefficients (P < 0.01) as the criterion in the process of item-total correlation.

*Content validity index* Content validity was defined as the proportion of experts who judged an item as content valid and as calculated using the content validity index (CVI). In this study, we assessed the CVI for each item (I-CVI). The scale was sent to five experts (three chronic disease experts and two nursing educators). The eligibility criteria for experts who were: (1) with the deputy director of the nurse or associate professor title or above; (2) bachelor degree or above; (3) have rich clinical nursing experiences in chronic disease management; (4) familiar with the development of measurement tools and methods for measuring the characteristics of psychological measurement. Content validity was assessed by each expert rating each item on a four-point Likert scale (1 = “uncorrelated” to 4 = ‘correlated”). The rating of each item was based on 2 criteria: (1) the applicability of content (referring to the appropriate level of expression and content with local culture and the study aims) and (2) clarity of phrasing (referring to the applicability of meaning in terms of description, clarity, understandability, and comprehension).The I-CVIs were calculated for each item for the CES. The minimum acceptable agreement score of the I-CVIs applied to this study was 0.7. The I-CVI scores were calculated as the number of scores greater than or equal to 4 divided by the number of experts. The panel was asked to make comments on individual items in relation to the accuracy, clarity, style, and cultural relevance of the translation. A panel-modified version was subsequently developed in this stage.

Exploratory factor analysis: Construct validity was assessed by a principal component factor analysis. The following criteria were used in order to obtain the best fitting structure and the correct number of factors: (a) eigenvalues greater than 1.0, (b) the percentage of total variance explained, and (c) factor loading cutoff at 0.30.

Internal consistency and test–retest reliability: Cronbach’s α was employed to estimate the internal consistency. Test–retest reliability were calculated using the interclass correlation coefficient. The final outcome of this step was the 44-item, 6-subscale Chinese version of the CES.

### Ethical considerations

The study was approved by research committee of the hospital. The research procedures and recruiting criteria were explained before contacted potential subjects. It was emphasized that participation was voluntary and could be withdrawn at any time, and subjects’ responses were considered anonymous and confidential. The researchers explained the risks and benefits of participation, and the patients’ right to refuse to participate without jeopardizing treatment. Participants were given verbal and written explanations of the purpose and design of the study. Methods and procedures of data collection, use and analysis, and usage were explained. Research data were then collected using a self-report questionnaire, where the patients were required to complete within 20 min.

### Data collection and analysis

All questionnaires were released by researchers, replied by patients independently. Completion of the questionnaires was checked immediately so as to confirm the effectiveness and completeness of the questionnaires.

Data were analyzed with SPSS for Windows Version 20.0. Descriptive statistics were used to summarize demographic characteristics. The Cronbach coefficient was used to assess the internal consistency of the CCES. The test–retest reliability of the baseline data and the 2-week follow-up were calculated using the interclass correlation coefficient. The construct validity of the CCES was examined using PCA by the varimax rotation method. The Kaiser–Meyer–Olkin (KMO) test was performed to examine the adequacy of data for PCA.

## Results

### Scale modification

Because of the linguistic and cultural differences in English and Chinese, the phrase of “elected town and government officials” in all 7 items were modified to “Healthcare Sector”. The item-total correlation coefficients of each item was 0.311–0.774, and all items were positively correlated with total score, correlation coefficient was statistically significant (P < 0.01). That was, the 44 items of the scale had a degree of differentiation, which could effectively identify the different patients’ reaction degree.

### Demographic data

Patients with chronic diseases came from three level-3 hospitals in Guangzhou, China, during the period from July 2015 to December 2015 by using convenience sampling method. A sample of 317 participants was recruited from 317 eligible patients (response rate of 100 %) for the study in which, 180 (56.8 %) were male patients, and 171 (53.9 %) were older than 60 years, and 77 (24.3 %) participants of the patients had a monthly income per person of more than 2000 RMB. Primary and secondary education were 121 participants (38.2 %) 0.146 (46.1 %) participants were living with chronic diseases more than 5 years. 68 participants (21.5 %) suffered from one chronic disease, and the remaining 249 patients (78.5 %) had two or more chronic diseases. Only 136 patients (42.9 %) attended health related lectures.

### Validity

For content validity, item scores of 4 or greater versus less than 4 were used to evaluate agreement by the content experts. All of the computed I-CVI values were greater than 0.8, and most were greater than 0.90. The average of CVI was 0.87, indicating adequate content validity.

The correlation between 6 dimensions, and the correlation between total score with the 6 dimensions were calculated to accessed validity of the CES and its dimensions. All dimensions were highly correlated, which were highly correlated with the overall, and the relationship between the degree of confidence, the dimension of Informed Confidence and Client–Provider Relationship were >0.9 (Table 2).

**Table 2:** Spearman correlation analysis between the Chinese version of CES scale and dimensions (n = 317)

Dimensions	Informed Confidence	Client–Provider Relationship	Social Advocacy	Awareness	Control	Client–Client Support
Informed Confidence	1					
Client–Provider Relationship	0.813**	1				
Social advocacy	0.805**	0.758**	1			
Awareness	0.764**	0.758**	0.731**	1		
Control	0.591**	0.539**	0.611**	0.483**	1	
Client–Client Support	0.604**	0.588**	0.537**	0.576**	0.313**	1
Total CES	0.943**	0.923**	0.890**	0.865**	0.658**	0.665**

** P < 0.01

The factorial structure assessment outcomes indicated a positive fit of the data for factor analysis. Bartlett’s test of sphericity was significant (χ^2^ = 7411.45, P < 0.01); the Kaiser–Meyer–Olkin measure of sampling adequacy was acceptable at 0.944. The principal component factor analysis extracted six eigenvalues greater than 1.0 with the cumulative variance of 54.91 %. The cutoff point of 0.30 was used to examine standardized factor loadings. Six factors emerged from the 44 items with all item loadings were greater than 0.30. Some items had factor loadings higher than 0.30 on more than 1 factor (Table 3). Most of them entries into their respective dimensions, which was in line with the theoretical model.

**Table 3:** Results of exploratory factor analysis

Items	Factors					
	1	2	3	4	5	6
I am confident managing the difficulties associated with my chronic health condition	0.413					
I am confident performing the skills necessary for managing my chronic health condition	0.710					
I know what helps me stay motivated to better manage my chronic health condition	0.522					
I am confident overcoming the obstacles that prevent me from achieving my health goals	0.710					
I know how to reach the goals I set about my health problems	0.629					
I know my capabilities and strengths necessary for managing my chronic health condition	0.329					
I am confident using my abilities to reach my health goals	0.501					
I know where to find support for my health care needs	0.360					
I am confident managing the pain associated with my chronic health condition	0.427					
I know my role versus the roles of my provider when solving problems associated with my chronic health condition	0.380					
I am confident performing therapeutic exercises as prescribed	0.541					
My provider listens attentively to me		0.471				
I collaborate with my provider in planning my health care regimen			0.499			
My provider encourages me to participate more in decisions related to my health care regimen		0.547				
My provider spends a fair amount of time during a visit		0.623				
My provider regards my knowledge and experience in taking care of my chronic health condition		0.402				
The positive attitudes of my provider motivates me to participate more in making decisions related to my health care regimen		0.460				
My provider supports my health care choices even if it contradicts with his/her recommendations		0.587				
I feel having a voice when interacting with my provider		0.391				
I am comfortable discussing my health concerns with my provider		0.803				
I am comfortable asking my provider questions related to my health problems		0.868				
I am comfortable expressing my feelings to my provider		0.817				
I consider myself an equal partner when interacting with my provider		0.596				
I often request additional health resources from my provider when needed		0.652				
I would identify problems related to my chronic health condition to Healthcare Sector for the benefit of all patients			0.511			
I would ask Healthcare Sector to consider including patients in the health policy process			0.750			
To improve the health care of peers, I would contact Healthcare Sector by writing them a letter			0.724			
I would state my opinion to HealthCare Sector for the benefit of all peers			0.487			
To improve the health care of peers, I would place a call to Healthcare Sector			0.300			
I would contact Healthcare Sector to expand health coverage for the alternative therapies			0.612			
I would ask Healthcare Sector to provide me with available community resources			0.699			
I would attend an event or a public meeting to discuss problems related to my chronic health condition			0.721			
I have the right to discuss my use of medication with my provider		0.460				
I would ask my provider to change my medication when it does not satisfy me				0.760		
I would talk to my provider if I feel that a wrong therapy is prescribed for me		0.357				
I am responsible for the decisions I make about my health		0.650				
I would talk to provider if I feel that a wrong procedure is to be performed on me		0.575				
I have the right to discuss my food preferences with my provider				0.505		
I would ask my provider to change the dietary regimen if it does not satisfy me		0.568				
I would stop a prescribed therapy that I am not satisfied with, even if it contradicts my provider’s recommendations					0.577	
I make the final decision regarding my health care					0.501	
I can refuse any treatment suggested by my provider if it does not satisfy me					0.416	
I share my health experiences with peers						0.432
I assist peers to manage their chronic health condition						0.617

### Reliability

Cronbach’s α coefficients were 0.956 for the CCES. 0.899 for Informed Confidence, 0.770 for Client–Provider Relationship, 0.797 for Social Advocacy, 0.753 for Awareness, 0.732 for Control and 0.698 for Client–Client Support, indicating very acceptable internal consistency reliability. The correlation of test–retest >0.7, Pearson correlation coefficient was 0.991, indicating good external consistency (Table 4).

**Table 4:** The reliability of the Chinese version of the CES scale

Dimensions	Chinese Cronbach’s α	English Cronbach’s α	Test–retest reliability
CES	0.947	0.97	0.991
Informed Confidence	0.899	0.94	0.978
Client–Provider Relationship	0.770	0.95	0.985
Social Advocacy	0.797	0.94	0.915
Awareness	0.753	0.90	0.982
Control	0.732	0.71	0.916
Client–Client Support	0.698	0.77	0.775

## Discussion

The present study provided evidence to support the 44-item CCES as a reliable instrument. This study strictly followed the principle of scale translation, and the experts we choose were familiar with the characteristics of psychological measurement method, with more than 10 years of clinical nursing or teaching experiences. Therefore, the quality of scale was assured. Results of this study showed that each item correlated highly with the total score, which suggests that the items in the CCES are relatively homogeneous and are measuring the same overall construct. The Cronbach’s α coefficients for the CCES was highly acceptable at 0.956, indicating satisfactory internal consistency of the scale. But the Cronbach’s α coefficients of six subscales was lower than those of the original scale, which meant that some items should be revised according to our national conditions and culture for further study, especially the subscales of Client–Client should add more items so the accuracy of the CCES to measure the empowerment level in China could be improved. Regarding the stability of the instrument, the test–retest reliability of the scale over 2 weeks from the current study was highly acceptable, with intraclass correlation coefficient scores for each item of this scale exceeding 0.80 and with no statistically significant differences in scale scores over a 2-week period.

Factor analysis revealed six factors that fit easily into the six dimensional model. These six factors explained 54.91 % of the total variance of the CCES Theoretically, the CES measures six constructs: Informed Confidence, Client–Provider Relationship, Social Advocacy, Awareness, Control, Client–Client Support. Factor loadings ranged from 0.329 to 0.868, which were acceptable. The factors extracted in this study were mostly consistent with the six original subscales in the CCES. However, our study indicated that the items of Awareness dimension were most entry Client–Provider Relationship dimension. It might because most Chinese patients did not fully recognized for their rights which cause they listen to their doctors. Our findings indicated that 44 items identified these 6 factors. As such, it means that the CCES can valid measure empowerment of Chinese patients with chronic disease.

Health care is the setting in which a high proportion of patients with chronic diseases are managed (Kettunen et al. 2001). Developing a valid and reliable measure of empowerment for use in this particular setting will assist in exploring the impact of empowerment in primary care and allow the measurement of the effects of interventions which aim to increase empowerment. An unpublished systematic review conducted by the authors found few instruments designed to measure empowerment in patients with chronic diseases and those that exist have been developed for particular chronic disease, such as diabetes (Anderson 2000), cancer (Suen 1998) and specific contexts, such as rehabilitation and self-help settings.

As empowerment is viewed as a priority by patients and professionals, there is consequent interest in improving levels of empowerment (Atak et al. 2008). Any systematic attempt to assess empowerment is dependent in part on the effective measurement of the concept. The overall findings of this study provide support for the construct validity of the CES scale as a measure of the ‘client empowerment’ construct. Being ‘empowered’ to manage chronic disease was demonstrated by six dimensions: (a) Informed-Confidence, (b) Client–Provider Relationship, (c) Social Advocacy, (d) Awareness, (e) Control, and (f) Client–Client Support. Through the six dimensions, this scale may be used in research studies to measure empowerment prior and post implementation of an empowering program with a specific sample of participant who live with chronic health conditions. For example, awareness items depicted the client’s awareness of their rights to discuss changes in the health care plan with the provider (Beaglehole 2008), and control items is the perceived ability to self-determine the course of a chronic condition and to choose among various options and alternatives (Wåhlin et al. 2006).

So, in summary, the Chinese version of the CES has the potential to measure and evaluate level of empowerment related to patient-empowerment concepts. It can be used to improve the outcomes of clinical-care services in China in relation to the level of perceptions of empowerment in clinical practice.

## Conclusion

To the best of our knowledge, this is the first study on an empowerment scale for the patients with chronic diseases in China. We conclude that the CCES has quite acceptable reliability and validity. Our findings confirm 6 factors in the CCES, which are informed confidence, client–provider relationship, social advocacy, awareness, control and client–client support. Our findings suggest that the CCES can be readily used by healthcare providers as a useful tool for assessing the empowerment level for patients with chronic diseases in China.

## Limitations

The study has some limitations which have to be pointed out. First, the participants were recruited by a convenience sampling method; thus, it may not be representative of the target chronic population. Another major limitation to this study population is selected on a voluntary basis, which would potentially introduce selection bias. Voluntary bias can be defined as the result of the fact that a particular sample can contain only those participants who are actually willing to participate in the study and who participate and find the topic particularly interesting are more likely to volunteer for that study, same to those who are expected to be evaluated on a positive level (Middleton 1997). Thirdly, the study was established on patients selected from a single city. Similar studies should be conducted in different geographic locales in China; and multigroup modeling should be conducted to examine invariance of the factorial structure, measurement parameters, and structural parameters of the CCES. Fourthly, our measurements were conducted at only a single point in time and, by clear inference, would not only be able to be used to reflect long-term exposure to various aspects or factors, which might be important influencers of CES, but also only internal consistency was assessed due to no demonstrated reliability of the Chinese CES in terms of stability over time. Fifthly, published standards for translation of health measurement scales recommend two independent translations, review by expert panel, two independent back-translations, and 2nd review by expert panel. However, due to language limitations, the measurement error was inevitable. In order to ensure that the translation procedures were robust, further studies should consider conducting some cognitive interviews to ensure the Chinese wording of the items was appropriate.

## Appendix 1

**Table:** The Client Empowerment Scale (CES)

Item number	Item statement	Strongly disagree	Disagree	Unsure	Agree	Strongly agree
1	I am confident managing the difficulties associated with my chronic health condition	1	2	3	4	5
2	My provider listens attentively to me	1	2	3	4	5
3	I would identify problems related to my chronic health condition to elected town and government officials for the benefit of all patients	1	2	3	4	5
4	I have the right to discuss my use of medication with my provider	1	2	3	4	5
5	I would stop a prescribed therapy that I am not satisfied with, even if it contradicts my provider’s recommendations	1	2	3	4	5
6	I am confident performing the skills necessary for managing my chronic health condition	1	2	3	4	5
7	I share my health experiences with peers	1	2	3	4	5
8	I collaborate with my provider in planning my health care regimen	1	2	3	4	5
9	I would ask my provider to change my medication when it does not satisfy me	1	2	3	4	5
10	I know what helps me stay motivated to better manage my chronic health condition	1	2	3	4	5
11	I would ask elected town and government officials to consider including patients in the health policy process	1	2	3	4	5
12	My provider encourages me to participate more in decisions related to my health care regimen	1	2	3	4	5
13	I am confident overcoming the obstacles that prevent me from achieving my health goals	1	2	3	4	5
14	To improve the health care of peers, I would contact elected town and government officials by writing them a letter	1	2	3	4	5
15	My provider spends a fair amount of time during a visit	1	2	3	4	5
16	I know how to reach the goals I set about my health problems	1	2	3	4	5
17	I would talk to my provider if I feel that a wrong therapy is prescribed for me	1	2	3	4	5
18	My provider regards my knowledge and experience in taking care of my chronic health condition	1	2	3	4	5
19	I know my capabilities and strengths necessary for managing my chronic health condition	1	2	3	4	5
20	I am responsible for the decisions I make about my health	1	2	3	4	5
21	I would state my opinion to elected town and government officials for the benefit of all peers	1	2	3	4	5
22	The positive attitudes of my provider motivates me to participate more in making decisions related to my health care regimen	1	2	3	4	5
23	I am confident using my abilities to reach my health goals	1	2	3	4	5
24	I make the final decision regarding my health care	1	2	3	4	5
25	My provider supports my health care choices even if it contradicts with his/her recommendations	1	2	3	4	5
26	I would talk to provider if I feel that a wrong procedure is to be performed on me	1	2	3	4	5
27	I know where to find support for my health care needs	1	2	3	4	5
28	To improve the health care of peers, I would place a call to elected town and government officials	1	2	3	4	5
29	I feel having a voice when interacting with my provider	1	2	3	4	5
30	I am confident managing the pain associated with my chronic health condition	1	2	3	4	5
31	I am comfortable discussing my health concerns with my provider	1	2	3	4	5
32	I have the right to discuss my food preferences with my provider	1	2	3	4	5
33	I know my role versus the roles of my provider when solving problems associated with my chronic health condition	1	2	3	4	5
34	I would contact elected town and government officials to expand health coverage for the alternative therapies	1	2	3	4	5
35	I assist peers to manage their chronic health condition	1	2	3	4	5
36	I am comfortable asking my provider questions related to my health problems	1	2	3	4	5
37	I am confident performing therapeutic exercises as prescribed	1	2	3	4	5
38	I would ask elected town and government officials to provide me with available community resources	1	2	3	4	5
39	I am comfortable expressing my feelings to my provider	1	2	3	4	5
40	I can refuse any treatment suggested by my provider if it does not satisfy me	1	2	3	4	5
41	I would ask my provider to change the dietary regimen if it does not satisfy me	1	2	3	4	5
42	I consider myself an equal partner when interacting with my provider	1	2	3	4	5
43	I would attend an event or a public meeting to discuss problems related to my chronic health condition	1	2	3	4	5
44	I often request additional health resources from my provider when needed	1	2	3	4	5

## Appendix 2

**Table:** 中文版患者赋权量表 (CCES)

项目序号	项目陈述	强烈不同意	不同意	不确定	同意	强烈同意
1	我有自信能解决和我的慢性疾病有关的困难。	1	2	3	4	5
2	医护人员会用心倾听我的话。	1	2	3	4	5
3	为了所有患者的利益,我会将有关我慢性疾病的问题提交给有关部门。	1	2	3	4	5
4	我有权利和我的医护人员讨论我的药物治疗使用情况。	1	2	3	4	5
5	我会停止不适合我的处方治疗,即使它违背了医护人员的建议。	1	2	3	4	5
6	我自信我能具备管理慢性疾病的必要技能。	1	2	3	4	5
7	我会和其他患者分享我的健康心得。	1	2	3	4	5
8	我会和医护人员合作规划我的健康护理方案。	1	2	3	4	5
9	当药物治疗方案不适宜我的时候,我会请求医护人员改变它。	1	2	3	4	5
10	我知道如何让自己保持乐观以更好的管理我的慢性疾病。	1	2	3	4	5
11	我会要求有关部门在制定卫生政策过程中将病人的意见纳入考量。	1	2	3	4	5
12	医护人员会鼓励我多参与决策我的健康护理方案。	1	2	3	4	5
13	我有信心克服那些阻止我实现我的健康目标的障碍。	1	2	3	4	5
14	为改善其他患者的医疗保健,我会写信建议有关部门。	1	2	3	4	5
15	医护人员会用足够多的时间进行查房。	1	2	3	4	5
16	我知道如何达到我为我健康问题设定的目标。	1	2	3	4	5
17	如果我觉得治疗有问题,我会和医护人员沟通。	1	2	3	4	5
18	医护人员认可我在照顾我的慢性疾病方面的知识与经验。	1	2	3	4	5
19	我知道我的能力和力量对管理我慢性疾病的重要性。	1	2	3	4	5
20	我对自己对健康所做的决定负责。	1	2	3	4	5
21	我会为了所有其他患者的利益向有关部门说出自己意见。	1	2	3	4	5
22	医护人员的积极态度会促使我更多地参与做出有关我的健康护理方案的决定。	1	2	3	4	5
23	我自信能用自己的能力达到我的健康目标。	1	2	3	4	5
24	我能对自己的健康问题做出最终决定。	1	2	3	4	5
25	医护人员支持我对自己医疗保健所做选择,即使和他/她的建议相矛盾。	1	2	3	4	5
26	如果我觉得将对我实施的治疗是错误的,我会和医护人员沟通。	1	2	3	4	5
27	我知道哪里可以满足我的医疗保健需求。	1	2	3	4	5
28	为了改善医疗保健服务,我会打电话给相关部门。	1	2	3	4	5
29	在和医护人员的交流中,我有一定的话语权。	1	2	3	4	5
30	我自信能管理自己慢性疾病相关的疼痛。	1	2	3	4	5
31	我能很自在地和医护人员讨论我的健康问题。	1	2	3	4	5
32	我有权利和医护人员讨论我的食物偏好。	1	2	3	4	5
33	我清楚在解决和我的慢性疾病有关的问题时,我自己和医护人员的所对应角色。	1	2	3	4	5
34	我会建议相关部门增加慢性疾病替代疗法的医疗支出。	1	2	3	4	5
35	我会协助其他患者管理他们的慢性疾病。	1	2	3	4	5
36	我能自在地询问医护人员关于自己健康方面的问题。	1	2	3	4	5
37	我自信能执行规定的锻炼治疗。	1	2	3	4	5
38	我会要求相关部门给我提供可用的社区资源。	1	2	3	4	5
39	我能自在地向医护人员表达我的感受。	1	2	3	4	5
40	如果觉得不适合我,我可以拒绝医护人员建议的任何治疗。	1	2	3	4	5
41	如果觉得不适合我,我会要求医护人员改变饮食方案。	1	2	3	4	5
42	在和医护人员的交互中,我认为自己是平等的伙伴关系。	1	2	3	4	5
43	我会参加讨论和我慢性疾病相关问题的活动或公众会议。	1	2	3	4	5
44	我常在有需要时向医护人员要求额外的健康资源。	1	2	3	4	5
